# Family Breakup Dynamics in a Promiscuous Solitary Mammal

**DOI:** 10.1002/ece3.72070

**Published:** 2025-09-03

**Authors:** Rick W. Heeres, Wisse Boot, Emile Delen, Miriam Tovar Heid, Martin Leclerc, Shane Frank, Alexander Kopatz, Fanie Pelletier, Henry Kuipers, Martijn J. A. Weterings, Andreas Zedrosser

**Affiliations:** ^1^ Department of Natural Sciences and Environmental Health University of South‐Eastern Norway Bø Norway; ^2^ Wildlife Management, Department of Animal Management Van Hall Larenstein University of Applied Sciences Leeuwarden the Netherlands; ^3^ Département Des Sciences Fondamentales & Centre D'étude de la forêt Université du Québec à Chicoutimi Chicoutimi Québec Canada; ^4^ Mammals Research Section Colorado Parks and Wildlife Fort Collins Colorado USA; ^5^ Norwegian Institute for Nature Research Trondheim Norway; ^6^ Département de Biologie Université de Sherbrooke Sherbrooke Québec Canada; ^7^ Wildlife Ecology and Conservation Group Wageningen University Wageningen the Netherlands; ^8^ Institute of Wildlife Biology and Game Management University of Natural Resources and Life Sciences Vienna Austria

**Keywords:** maternal investment, movement behavior, parental care, solitary mammal, Sweden, *Ursus arctos*

## Abstract

Family breakup dynamics in mammals can be complex due to competing interests between parents and offspring. Parents need to balance their own as well as their offspring's fitness through either terminating care early or extending care. Yet, males can disrupt this trade‐off as they may force females to focus on future litters by separating or killing offspring, especially in species where sexually selected infanticide occurs. Here, we investigated the family breakup dynamics in brown bears ( *Ursus arctos* ) by using GPS relocation data from 144 individuals (114 unique individuals: 23 mothers, 49 offspring, and 42 adult males) in southcentral Sweden. We explored the movement of mothers, their offspring, and adjacent adult males to gain insights into the factors influencing family breakup. Our findings indicate that females with 2‐year‐olds tend to separate before the mating season, whereas females with yearlings typically experience breakups during the mating season. Our results show that females accompanied by yearlings increased their movement speeds 2 weeks before the family breakup. The movement speed of the families that separated was two to three times higher compared to families that remained together. Furthermore, males associated with family groups before and during the mating season. Several associations during the mating season between adult males and family groups occurred on the same day that the family broke up. The increased space use makes the family group more conspicuous on the landscape; this likely increases the detection probability by a male and increases the chance of family breakup. Maternal care tactics can influence both female and offspring fitness, and here we provided additional evidence of the interplay between female and adult male behavior in terminating care in a solitary carnivore.

## Graphical abstract

Parental care is vital for the survival and development of offspring, yet family breakup dynamics can be complex due to competing interests between parents, neighboring conspecifics, and offspring. We provided additional evidence of the interplay between mothers, offspring, and adult male behavior in terminating maternal care.

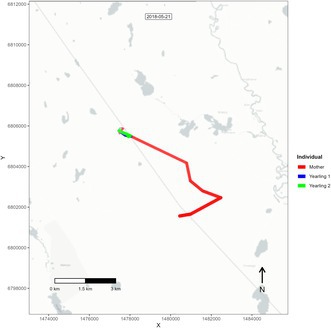

## 1 Introduction

Parental care is crucial for the survival and development of offspring in mammals (Clutton‐Brock 1991; Clutton‐Brock et al. 1982). Parents provide a social and safe environment where offspring learn essential knowledge and skills related to shelter, foraging, predation, and conspecifics (Engebretsen et al. 2024; Mateo 2009). For example, hunting techniques in canids and felids are passed on to offspring during foraging excursions with one or both parents (Bekoff et al. 1984). The quality and duration of parental care can influence the way offspring develop, adjust to their environment, and reproduce successfully later in life (Alberts 2019; Nowak 2000). However, parents need to balance their own as well as their offspring's fitness and commonly trade off between current and potential future reproduction. This trade‐off can lead to conflicts between parents and offspring regarding the length of the parental care (Trivers 1972).

In most mammals, males provide little to no parental care. Thus, parental care usually is exclusively provided by females (Alonzo and Klug 2012; Bateson 1994; Shuster and Wade 2003). In some mammals, females cannot remate until their current offspring are weaned, abandoned, or dead (Lee 1996; Wolff and MacDonald 2004). The separation of offspring stops mammary stimulation and lactation, which would otherwise suppress hormone production (e.g., prolactin) inhibiting a female from getting into estrus (Asa 2012). Early separation can increase the number of offspring a female can produce in her lifetime, but early weaning of offspring may lower their survival due to limited experience in foraging or social skills (Klug and Bonsall 2014; Lee et al. 1991; Trivers 1972; Webb et al. 1999). However, early separation may also expose these individuals to other challenges including a high predation risk, high energetic demands, and competition with conspecifics (Clutton‐Brock et al. 1985). Environmental factors such as resource availability, but also life history factors such as litter size as well as offspring sex and size can affect the duration of maternal care (Andersen et al. 2000; Balme et al. 2017; Johansson et al. 2021; Van de Walle et al. 2021). In general, higher resource abundance (i.e., the resource limitation hypothesis; Lee et al. 1991; Wilsterman et al. 2024) and smaller litter sizes are expected to shorten the duration of maternal care, while offspring sex (i.e., the sex‐allocation hypothesis; Hewison et al. 2005; Moore et al. 2015), larger litter size, and smaller offspring are expected to increase maternal care duration (Van de Walle et al. 2021). Before family separation (i.e., the moment that mothers and offspring go their separate ways), females may exhibit behaviors such as aggression or increased avoidance towards their offspring to initiate the breakup (Trivers 1974). In cases of avoidance, the movement behavior (e.g., daily travel distance) of females before separation could shed light on female‐induced breakup dynamics. However, the process of family separation might not be a process that involves only the female and her offspring, as adult males vying for mating opportunities may play an important role in causing or initiating separation (Dahle and Swenson 2003b; Packer and Pusey 1983; Pusey and Packer 1994; Swenson, Sandegren, et al. 2001). Males may play a particularly important role in species with sexually selected infanticide (SSI; Agrell et al. 1998; Hrdy 1979) in which females enter estrus shortly after offspring loss or separation, and males therefore benefit from breaking up family groups (Dahle and Swenson 2003b; Steyaert et al. 2014). Consequently, investigating male presence and movement behavior during family breakup can inform us on how social associations may shape a parent‐offspring conflict.

The brown bear ( *Ursus arctos* ) is a solitary large carnivore with a promiscuous mating system (Steyaert et al. 2012). The mating season lasts from early May to mid‐July (Heeres et al. 2024; Spady et al. 2007; Steyaert et al. 2012). Family breakups generally occur just before or during the first weeks of the mating season (Dahle and Swenson 2003b). For female brown bears in Scandinavia, the duration of maternal care is either 1.5 or 2.5 years (Dahle and Swenson 2003a; Van de Walle et al. 2021). Litter size and yearling body mass are important determinants of the short (1.5 years) and long (2.5 years) maternal care tactics in this population (Van de Walle et al. 2021). To initiate separation from offspring, females are presumed to alter their movement behavior to find mates (Dahle and Swenson 2003a, 2003b), and males have previously been hypothesized to play an important role in initiating family breakups (Dahle and Swenson 2003b). Until now, only the change in movement behavior after the loss of cubs‐of‐the‐year (Steyaert et al. 2014) and the habitat selection of females with dependent offspring before and after breakups (Van de Walle et al. 2019) have been investigated. However, by understanding female reproductive tactics and movement behavior, as well as the influence of males on these tactics, we can determine if changes in female space use correlate with maternal care termination or continuation. The study of reproductive tactics in the wild is challenging; however, in this study, we have all data components (i.e., mother, offspring, adult males) affecting family breakups of brown bears, which allows us to gain new insights into this crucial but rarely documented process.

The objective of this study was to examine the movement behavior of family groups, including mother and offspring, and adult males during the mating season in relation to family breakup dynamics in a solitary‐living mammal. We used GPS relocation data from 23 unique mothers (29 bear‐years; because some females were followed during several years and they had offspring), 42 unique males (75 bear‐years), and 49 unique offspring (1.5 and 2.5 years old) brown bears during the mating season in southcentral Sweden. We determined family breakup dates based on family movement behavior. We contrasted the daily travel distance of mothers before breakups with mothers that remained with their offspring. In addition, we determined male spatial and temporal proximity to family groups to investigate their potential role in family breakups. In American black bears ( *Ursus americanus* ) females with 2‐year‐olds instigate family breakup before the mating season (Clevenger and Pelton 1990). Thus, we predicted that (1) females accompanied by 2‐year‐olds should breakup earlier than females accompanied by yearlings. Parental care might be biased to either male or female offspring (Trivers and Willard 1973). We therefore expected that the breakup dates might differ between the sexes of the offspring, as males tend to disperse earlier than females (Zedrosser et al. 2007). Thus, we predicted that (2) male offspring separated from their mothers earlier than female offspring in both the yearling and 2‐year‐old age groups. Females accompanied by yearlings focusing on future reproduction should show different movement behaviors during the mating season compared to females that remained with their yearlings. Also, females that stay with their yearlings for an additional year are expected to avoid potentially infanticidal males, that is, males other than the ones they mated with the previous mating season (Dahle and Swenson 2003b; Heeres et al. 2025; Swenson, Dahle, and Sandegren 2001), and adjust their movement behavior accordingly. The change in behavior may mainly be displayed by reduced movement speed and changing habitat selection (Heeres et al. 2025; Steyaert et al. 2016; Van de Walle et al. 2019). Therefore, we predicted that (3) females experiencing a family breakup have higher daily travel distances before the breakup compared to females that remained with their offspring. Families including either yearlings (1.5 years old) or 2‐year‐olds (2.5 years old) can breakup before or at the beginning of the mating season (Dahle and Swenson 2003b). Lastly, in several bear species, male‐initiated family breakups have been documented (Dahle and Swenson 2003b; Ternent and Garshelis 1998). Yet, the movement dynamics before and following male interference on both the family and male have not been described in detail before. We therefore provide anecdotal observations from males interacting with family groups containing yearlings and 2‐year‐olds.

## 2 Material and Methods

### 2.1 Study Area

The study area is approximately 13,000 km^2^ and located in south‐central Sweden (61° N, 15° E). The area features relief ranging from 100 to 800 m above sea level, and is largely composed of bogs, lakes, and intensively managed coniferous forests. The mean temperatures in January and July are −7°C and 15°C, respectively (Bischof et al. 2018) and snow cover usually lasts from late October until early May. Human settlements are primarily found in the northern and southern parts of the study area. There are some high‐traffic roads (0.14 km/km^2^), but cabins and low‐traffic paved roads are scattered throughout the area (0.3/km^2^ and 0.7 km/km^2^, respectively; Martin et al. 2010). Human activity is higher during summer and fall due to hunting or berry and mushroom picking seasons (Ordiz et al. 2011). Brown bear population density in the study area is estimated at approximately 23 bears per 1000 km^2^ (Bischof et al. 2019). Brown bears in Sweden are legally hunted from 21 August to 15 October or until quotas are filled, and around 10% are harvested from the total population annually (Bischof et al. 2019).

### 2.2 Bear Individual Characteristics and Monitoring

The Scandinavian Brown Bear Research Project (SBBRP) follows a strict capture protocol (Graesli et al. 2025). Bears were darted from a helicopter using a remote drug delivery system (Dan‐Inject, Børkop, Denmark) and GPS‐collared (GPS Plus; Vectronic Aerospace GmbH, Berlin, Germany) on an annual basis. The collars were programmed to relocate all individuals every hour. See Graesli et al. (2025) for more details on captures and handling. All aspects of animal capture and handling were approved under an ethical permit by the Uppsala Ethical Committee on Animal Experiments (Dnr 5.8.18‐03376/2020). Our capture permit was provided by the Swedish Environmental Protection Agency (NV‐01278‐22).

We had access to location data of 29 families, including 29 adult females (i.e., mothers; 23 unique females) and 49 offspring (yearlings: *n* = 21, 2‐year‐olds: *n* = 28) during their active period (i.e., excluding hibernation) between 2007 and 2021. Location data of 42 unique adult males (> 3 years old; 75 bear‐years) were available as well. The age (e.g., year of birth) of most bears was known because they were captured as part of a family group. For bears not followed from birth, a premolar tooth was extracted for age determination (Matson et al. 1993). Female reproductive status was determined at capture or via visual observations from a helicopter several times per year (Van de Walle et al. 2021). These visual observations also made it possible to detect if family groups had broken up during the year. Based on the age of the offspring and the occurrence of a family breakup, we defined three states: (1) *yearling breakup*; females with yearlings where a breakup occurred, (2) *yearling family*; females with yearlings that stayed together until the next hibernation event, and (3) *2‐year‐old breakup*; females with 2‐year‐olds where a breakup occurred.

### 2.3 Breakup Date

**FIGURE 1:**
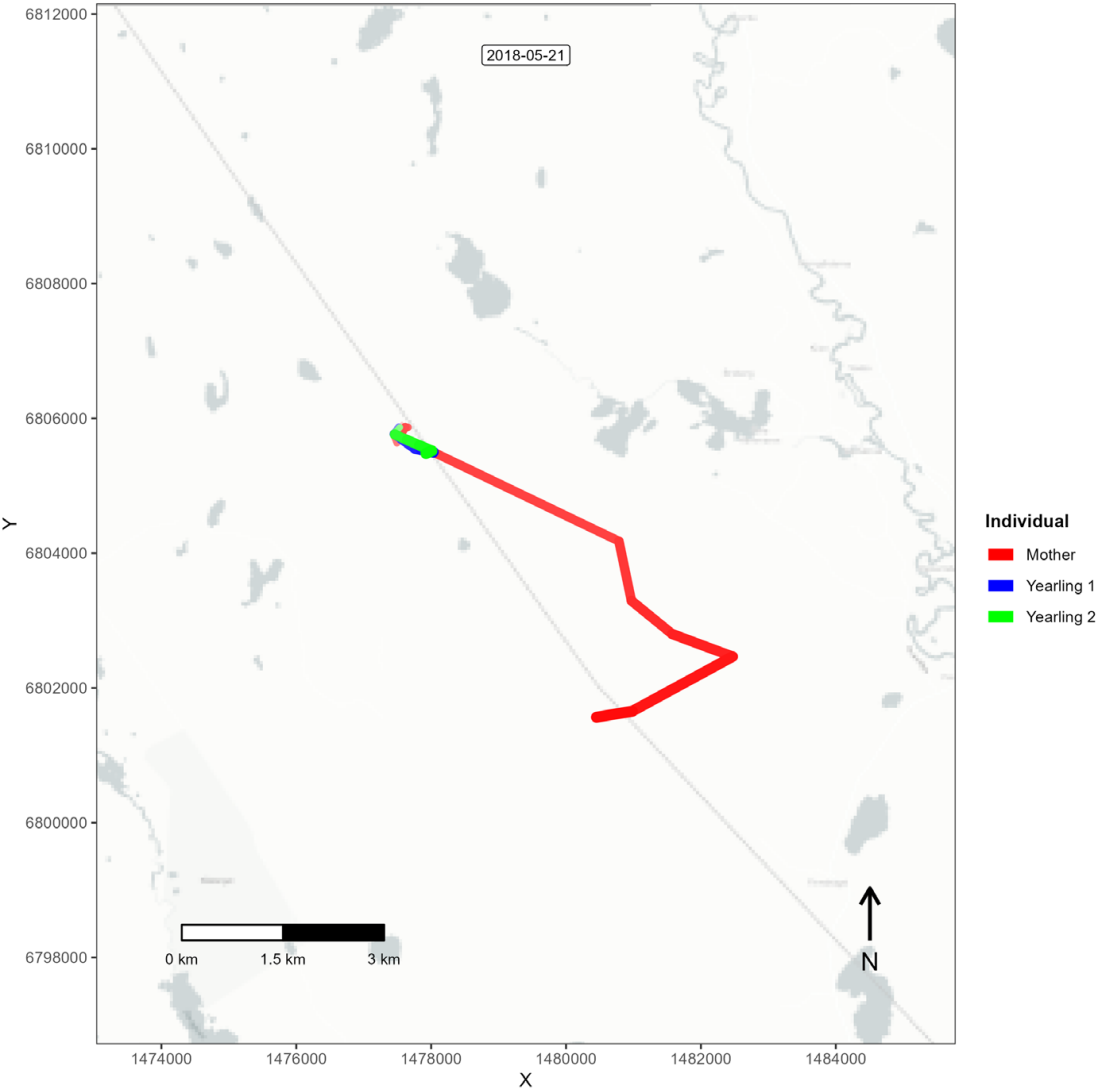
Animation, created by using *moveVis* package in R, showing the movement of a family including a mother (red line) and her two yearlings (green and blue lines) based on GPS location data in southcentral Sweden. The moment that the red dot and the green/blue dots separate shows the family breakup event.

Initially, a family breakup date was determined when a mother and her offspring were separated by more than 500 m (Lee and Vaughan 2004) for at least 24 h. However, this threshold proved inaccurate, as there were numerous instances where offspring and mothers were found further than 500 m for a day but reunited again for an extended period (e.g., several weeks). Therefore, to achieve a more accurate determination of the breakup date, we visually inspected the movement trajectories (Video 1, Figure 1) of family groups before and during the mating season using the *moveVis* package (Schwalb‐Willmann et al. 2020) in R 4.4.2 (R Development Core Team 2024). These movement trajectories allowed us to confirm that no reassociations (i.e., within 500 m for at least a week) of the offspring and the mother occurred during the mating season. We identified the breakup date per offspring, making it possible to check for sex‐related differences in breakup timing.

**VIDEO 1:** Video content can be viewed at https://onlinelibrary.wiley.com/doi/10.1002/ece3.72070.

### 2.4 Female Movement Before Breakups

The *amt* package (Signer et al. 2019) was used to calculate the hourly travel distance and the total number of relocations per day (i.e., successful GPS fixes) for the females that either remained or separated from their yearlings. Once the breakup dates were set based on the visualization of the movement path of the family, we selected the movement data 14 days before the breakup. For the females that did not experience a breakup, we selected the data 14 days before the median breakup date (May 26: day of the year 146). Additionally, we determined the daily travel distance for all days that had at least 20 relocations to avoid underestimation of daily travel distances. Overall success rate of relocations in our study area is above 94% (Leclerc et al. 2016).

### 2.5 Adult Male Presence at Breakups

To investigate the interference of males in relation to family breakups, we first identified if the family group encountered a GPS‐collared male before or during the mating season. We calculated all pairwise distances between mothers and adult males utilizing hourly GPS relocations using the *spatsoc* package (Robitaille et al. 2019). We then used a distance threshold of 200 m (including a 3‐min temporal tolerance) to determine social associations (i.e., two or more individuals within 200 m at the same time; Heeres et al. 2024). The dates of a family breakup and all the associations a family had with an adult male were compared to determine if an adult male interfered in the family breakup. If an adult male was spatiotemporally associated with the family group during the day of the breakup, we investigated his and the family's movement patterns visually and determined how many times the male approached the family before the mating season and if the male was close to the family just before the family breakup.

### 2.6 Statistical Analysis—Breakup Date

We investigated if the breakup dates of family groups were different between the offspring age groups and sexes with the help of Generalized Linear Mixed Models (GLMM), fitted with the *glmmTMB* package (Brooks et al. 2017), with a Poisson distribution (i.e., slightly skewed). We included the family group ID nested in mother ID as a random intercept to control for pseudoreplication (Millar and Anderson 2004). The *DHARMa* package (Hartig 2022) was used to evaluate model fit. We evaluated different candidate models (Appendix S1, Table S1) and determined the most parsimonious one with the corrected Akaike Information Criteria (AICc; *AICcmodavg* package: Mazerolle 2023).

### 2.7 Statistical Analysis—Mother Movement Before Breakups

We investigated the movement patterns of mothers with yearlings before (14 days) family breakup using the *mgcv* package (Wood 2017) and fitted a hierarchical generalized additive mixed model (HGAM; Pedersen et al. 2019) with a Gamma (log link) distribution. The model included the group classification (yearling family = 0, yearling family breakup = 1) to identify if there was a significant difference in daily travel distances between the two groups. Additionally, we included the number of successful fixes (relocations ≥ 20) as an offset variable to control for its potential influence on the daily total travel distance. We used a GAMM as we expected a non‐linear pattern (Wood 2017) and we used the number of days before the breakup date as a covariate in the smoothers (Pedersen et al. 2019). We included an AR1 temporal autocorrelation structure by using the day of the year combined with the individual ID (Mitchell et al. 2019; Zuur et al. 2009). We also added the unique ID of the mother as a random variable to consider individual differences of adult females. The model fit was validated using both the *mgcv* and *mgcViz* packages (Fasiolo et al. 2020).

## 3 Results

### 3.1 Breakup Dates

**FIGURE 2:**
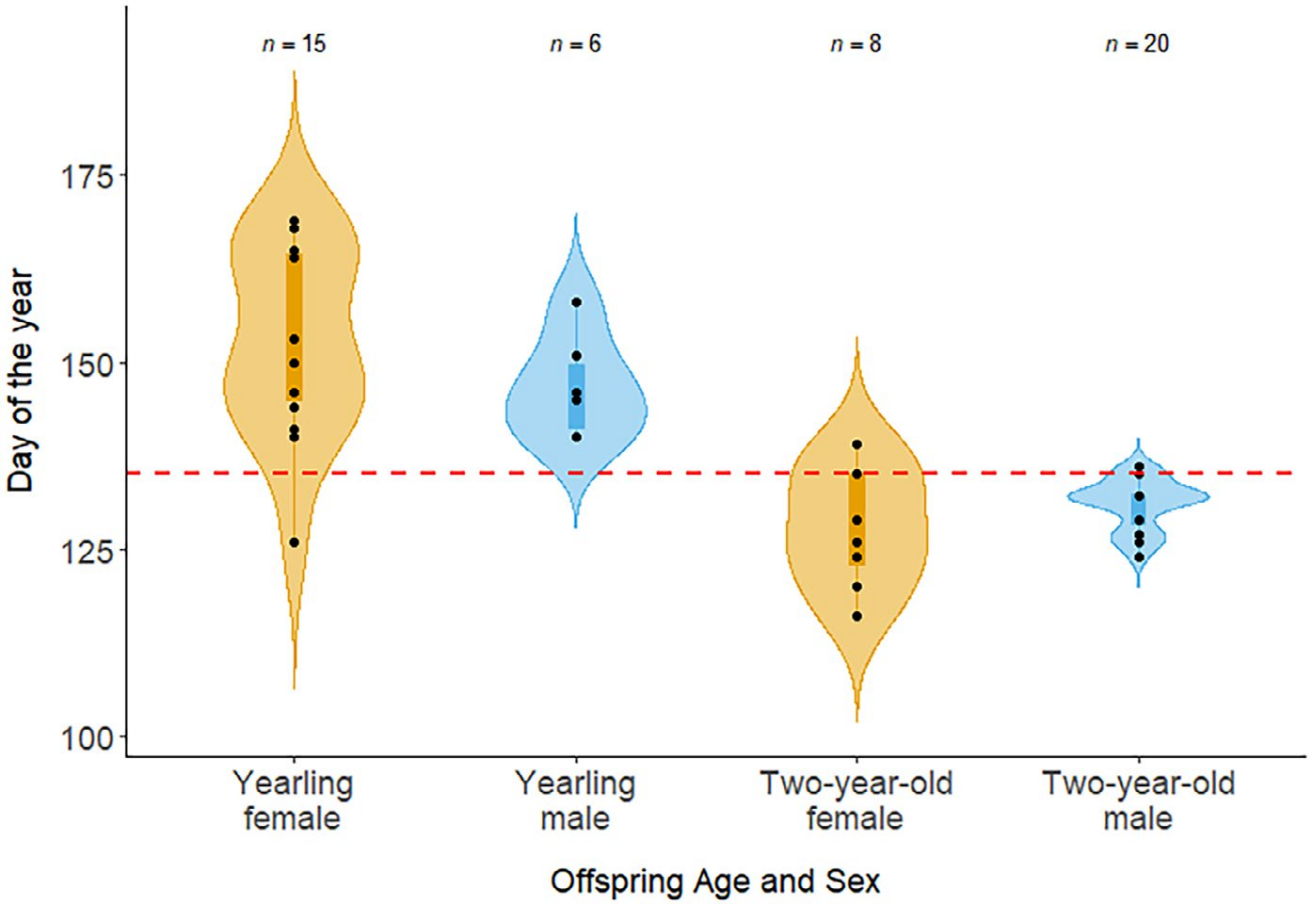
Breakup dates for brown bear family groups (*n* = 29) southcentral Sweden (2007–2021), including one or more yearlings (*n* = 15, individuals = 21) or 2‐year‐olds (*n* = 14, individuals = 28). Breakup dates were conjointly determined by spatiotemporal thresholds and animations. The mating season starts around the day of the year 135 (May 15), indicated by the horizontal red dotted line.

We found a significant difference (Figure 2) in the breakup dates between family groups with yearlings (*n* = 21) and 2‐year‐olds (*n* = 28) using a GLMM model. The most parsimonious model only included the age of the offspring (Appendix S1: Table S1). Families with 2‐year‐olds broke up earlier in the season in comparison to families with yearlings (*β* = −0.82 (log‐scale), *p* < 0.001; Table 1 and Appendix S1: Table S1 & Figure S1). Therefore, the sex of the offspring did not influence the timing of family breakups for yearlings and 2‐year‐olds.

**TABLE 1:** The median and range of the breakup date (day of the year; doy) per age and sex class of offspring of brown bears (family groups: *N* = 29) in southcentral Sweden (2007–2021). Breakup dates were conjointly determined by spatiotemporal thresholds and spatial animations.

Age	*n*	Sex	Samples	Median date (doy)	Range (min‐max)
Yearling	21	Female	15	150	126–169
		Male	6	146	140–158
2‐year‐olds	28	Female	8	128	116–139
		Male	20	132	124–136

### 3.2 Movement Behavior of Mothers Before Break Ups

**FIGURE 3:**
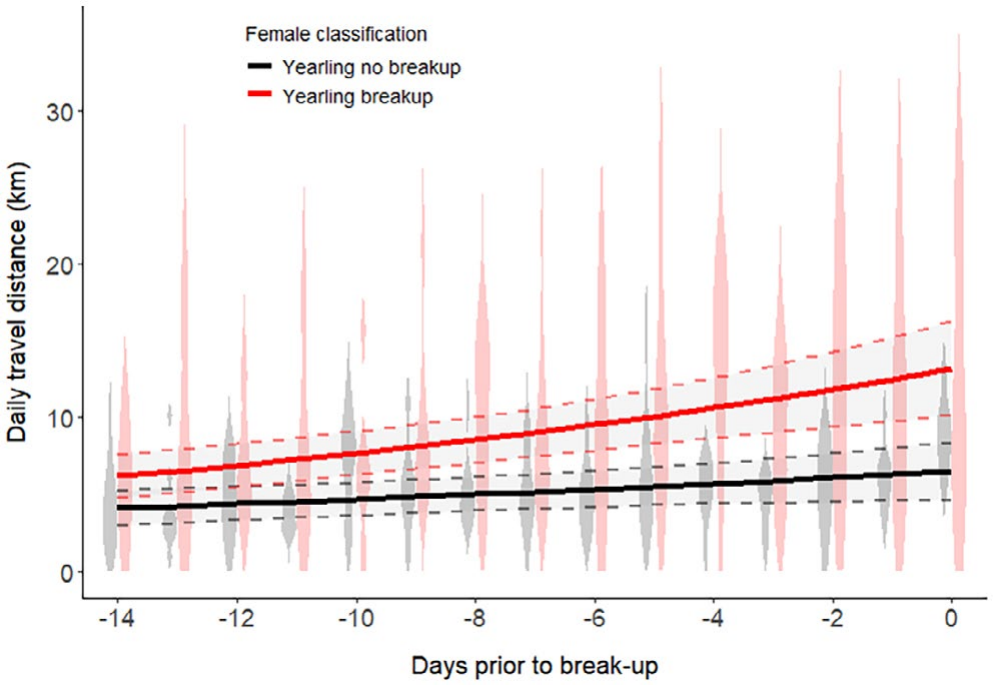
The change in daily travel distance (km) by female brown bears accompanied by dependent offspring in southcentral Sweden (2007–2021) across 14 days prior to a family breakup. The lines represent the mean daily travel distance of females with yearlings experiencing a breakup (*n* = 15; red line) and females with yearlings without a breakup (*n* = 10; black line). For families without breakup, we selected the 14 days prior to the median breakup date (doy 146; May 26). The shaded areas and dotted lines represent the 95% confidence bands. The violin plots in the background show the raw daily travel distance data per female classification.

We found that females who separated from their yearlings (*n* = 15) had significantly higher daily travel distances before the breakup in comparison to females that did not breakup (*n* = 10) during the same period (*β* = 0.545, *p* < 0.001; Figure 3). There was a steady increase in the daily travel distance over the 14 days before the family breakup.

### 3.3 Male Presence During Breakups

**FIGURE 4:**
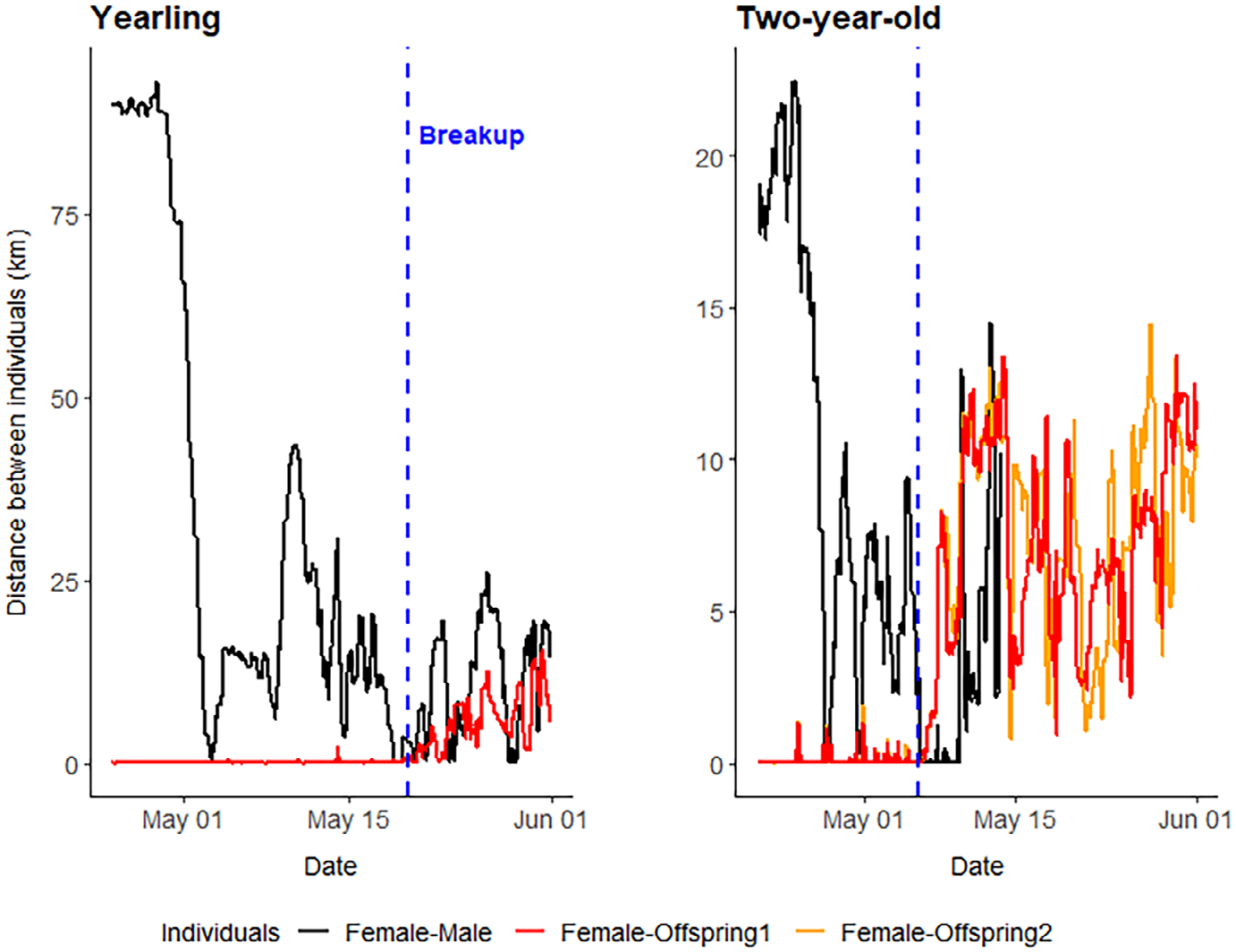
The distance between mothers and offspring (red line) and mothers and adult male (black line) prior to and during the first weeks of the mating season for two families. The start of the mating season is around May 15. The blue dotted line is the breakup date of the family conjointly determined by using spatiotemporal thresholds and animations. All data were collected in southcentral Sweden (2007–2021).

Our data showed that 11 of 29 families (37.9%) did not associate with a GPS‐collared male before or during the mating season. We observed 14 of 29 families (48.3%) that associated with GPS‐collared males before and/or during the mating season, but not on the day of a family breakup, and 4 of 29 females (13.8%) associated with a GPS‐collared male during the day of the family breakup (*n* = 1 family with yearlings; *n* = 3 family with 2‐year‐olds). After the initial association between the female with yearlings and an adult male, followed by family breakup, we observed that males did not always stay close to the female but occasionally moved away and came back in proximity to the female over a few weeks, followed by a longer association close to or during the mating season (Figure 4 and Appendix S2: Male Family Breakup 1). We also observed one occasion where a male visited a family three times before the mating season; however, the family reunited shortly after the first two visits. The male was in proximity during the family breakup a week later and remained with the female for several days after (Figure 4 and Appendix S2; Male Family Breakup 2).

## 4 Discussion

The objective of this study was to examine the movement behavior of mothers, offspring, and adult males before and during family breakups in brown bears. We found, as predicted, that females with yearlings' primarily break up during the mating season, whereas females with 2‐year‐olds break up before the mating season. We also showed that the breakup date was not correlated to the sex of the offspring, neither for yearlings nor 2‐year‐olds. As predicted, females that broke up from their yearlings traveled significantly longer daily distances before breakup, in contrast to females that remained with their yearlings. Lastly, we observed few occasions (4 out of 29) during which a GPS‐collared male was present on the day of the family breakup. However, GPS‐collared males visited family groups and lone females (if family breakup already occurred) before the mating season regularly.

The timing of separation from offspring can lead to parent‐offspring conflicts (Sunde 2008), especially in species where females can only reproduce once their current offspring have weaned or dispersed (Trivers 1974). In American black bears, it has been suggested that it is the mother who initiates a family breakup (Clevenger and Pelton 1990; Rogers 1987). Physiological factors, such as ending lactation or entering estrus, might urge the female to break up with the offspring (Asa 2012). In brown bears, it seems that the majority of breakups occur at the beginning of the mating season (Dahle and Swenson 2003b). However, our results showed a further differentiation of this timeline, with females with 2‐year‐olds generally breaking up before the mating season, while most females with yearlings' break up during the mating season. Breakups with yearlings might result in more pronounced parent‐offspring conflict, as some females show increasing movement distances, which are likely to result in separation from their offspring. This increase in travel distances likely tires out the offspring (i.e., increase energy expenditure) but might also lead to a higher probability of detection by roaming males, likely indirectly promoting male participation in family separation. Additionally, the sex‐allocation hypothesis (Trivers and Willard 1973) is based on the idea that parental care might be biased to either male or female offspring (Hewison et al. 2005; Koskela et al. 2009; Moore et al. 2015) and this could potentially lead to different breakup dates for offspring of the same litter based on their sex. However, we did not find a difference in the breakup date between the sexes for either yearlings or 2‐year‐olds. This is likely because families that are broken up by adult males cause all offspring to separate at the same time, making it difficult to identify clear sex‐specific breakup dates for many species in which sexually selected infanticide is common.

After emerging from their den, males utilize a “roam‐to‐mate” behavior to detect and approach females (Dahle and Swenson 2003c). We found anecdotal evidence of males approaching family groups before the mating season potentially to explore the reproductive status of the female. This is also common among other species, such as rodents and ungulates (Eisenberg and Kleiman 1972; Grau 1976; Johansson and Jones 2007; Weir and Rowlands 1973). This suggests that males seem to prepare for the mating season and investigate which females are “available” in their home range. This also helps to identify family groups and additional opportunities to reproduce by separating the mother from their offspring. The advantage for adult males of breaking up family groups is that they “initiate” mating opportunities that otherwise might not have been available (Lukas and Huchard 2014). Sexually selected infanticide is a common male mating tactic in brown bears (Bellemain et al. 2006; Steyaert et al. 2012) and is only successful when the offspring are killed during the mating season, which leads to the female entering estrus within several days (Steyaert et al. 2014). Therefore, males could have an incentive to break up family groups, irrespective of the offspring's age, and thereby gain additional mating opportunities.

We did not have data regarding the physiological state (e.g., hormone levels) of females before the family breakups. Therefore, we are restricted to identifying changes in behavior, and here we use movement as a proxy for a female's motivation to break up with her offspring. In addition, we only observed 4 occasions where males were present near the family group at the time of the family breakup. However, this may be related to the small sample size of GPS‐collared adult males per year. In addition, family groups may have interacted with uncollared males during the time of the breakup. Thus, we likely underestimate the impact of males on family breakup dynamics. Future studies should try to disentangle social dynamics before associations for not only family groups with adult males but also other groups (e.g., lone females). This could show that social information is gained not only with spatiotemporal associations but also other factors such as the “scentscape” (Clapham et al. 2012; Finnerty et al. 2022; Marin et al. 2021; Sergiel et al. 2017).

Maternal care is directly linked to the early survival of offspring and influences future reproduction by the mother (Mateo 2009). Therefore, it is crucial to understand which aspects influence the duration of maternal investment (Balme et al. 2017; Johansson et al. 2021; Van de Walle et al. 2021), but also the fitness benefits of either terminating or extending care for both the mother and offspring (Van de Walle et al. 2018). We do know that female brown bears benefit from providing longer maternal care, which may be related to artificial selection caused by the harvest regime (Van de Walle et al. 2018). However, it would be interesting to see if the duration of maternal care, short versus long maternal care (Zedrosser et al. 2013), also benefits the offspring (e.g., increased fitness, early primiparity). This would make it possible to evaluate the costs and benefits of varying maternal care tactics and investigate their influence on population dynamics and demography.

## Author Contributions

**Rick W. Heeres:** conceptualization (equal), data curation (lead), formal analysis (lead), methodology (equal), writing – original draft (lead), writing – review and editing (lead). **Wisse Boot:** conceptualization (supporting), formal analysis (supporting), methodology (equal), writing – review and editing (supporting). **Emile Delen:** conceptualization (supporting), formal analysis (supporting), methodology (equal), writing – review and editing (supporting). **Miriam Tovar Heid:** conceptualization (supporting), formal analysis (supporting), methodology (equal), writing – review and editing (supporting). **Martin Leclerc:** formal analysis (supporting), writing – review and editing (equal). **Shane Frank:** formal analysis (supporting), writing – review and editing (equal). **Alexander Kopatz:** project administration (equal), writing – review and editing (equal). **Fanie Pelletier:** funding acquisition (equal), supervision (equal), writing – review and editing (equal). **Henry Kuipers:** conceptualization (equal), formal analysis (supporting), methodology (equal), writing – review and editing (equal). **Martijn J. A. Weterings:** conceptualization (equal), formal analysis (supporting), methodology (equal), writing – review and editing (equal). **Andreas Zedrosser:** funding acquisition (equal), supervision (equal), writing – review and editing (equal).

## Conflicts of Interest

The authors declare no conflicts of interest.

## Supporting information


**Table S1:** Model selection for the Poisson GLMM models using the family breakup dates (*n* = 49) in Sweden (2007–2021). “Model” identifies the included variable(s), the “AICc” is the AICc estimate per model, the delta AICc, the “*k*” indicates the number of parameters, and “*LogLik*” represents the log likelihood. The most parsimonious model is shown in **bold**. The models including two‐way interactions are indicated by the (x) between variables in the model information. All models included a nested random intercept including mother‐ and family‐ID.
**Figure S1:** Predicted breakup date range in relation to the age of the offspring (1.5‐year‐old yearlings = orange and 2.5‐year‐old two‐year‐olds = blue) in southcentral Sweden (2007–2021). The prediction plot is based on the most parsimonious model (Table S1), only including the age of the offspring as a fixed variable and a nested random effect of mother‐ and family‐ID. The black dots represent the raw data (*n* = 49).

## Data Availability

R code and data that supports the findings of this study are available on our universities' USN Research Data Archive, here: https://doi.org/10.23642/usn.29225816. Additionally, the RAW GPS data that was used to create the data files for this study can be found here: https://doi.org/10.23642/usn.24949185.v2.
