# Supplementary material for: Family Breakup Dynamics in a Promiscuous Solitary Mammal
Source: Ecol Evol. 2025 Sep 3;15(9):e72070. doi: 10.1002/ece3.72070 (PMC12408101; doi:10.1002/ece3.72070)
Supplement: Supplementary file 1 — Table S1: Model selection for the Poisson GLMM models using the family breakup dates (n = 49) in Sweden (2007–2021). “Model” identifies the included variable(s), the “AICc” is the AICc estimate per model, the delta AICc, the “k” indicates the number of parameters, and “LogLik” represents the log likelihood. The most parsimonious model is shown in bold. The models including two‐way interactions are indicated by the (x) between variables in the model information. All models included a nested random intercept including mother‐ and family‐ID. Figure S1: Predicted breakup date range in relation to the age of the offspring (1.5‐year‐old yearlings = orange and 2.5‐year‐old two‐year‐olds = blue) in southcentral Sweden (2007–2021). The prediction plot is based on the most parsimonious model (Table S1), only including the age of the offspring as a fixed variable and a nested random effect of mother‐ and family‐ID. The black dots represent the raw data (n = 49). [file ECE3-15-e72070-s001.docx]

**Supplementary Materials**

**Appendix 1 – GLMM analysis breakup dates**

Table S1: Model selection for the Poisson GLMM models using the family breakup dates (*n* = 49) in Sweden (2007 – 2021). “Model” identifies the included variable(s), the “AICc” is the AICc estimate per model, the delta AICc, the “*k*” indicates the number of parameters, and “*LogLik*” represents the log likelihood. The most parsimonious model is shown in **bold**. The models including two-way interactions are indicated by the (x) between variables in the model information. All models included a nested random intercept including mother- and family-ID.

| Model | AICc | ΔAICc | *k* | *LogLik* |
| --- | --- | --- | --- | --- |
| **Age** | **329.13** | **-** | **2** | **-161.3** |
| Age + Sex | 331.30 | 2.17 | 3 | -161.2 |
| Age x Sex | 333.14 | 4.01 | 4 | -160.9 |
| Sex | 352.71 | 23.58 | 2 | -173.1 |
| Null (intercept-only | 590.56 | 261.43 | 1 | -294.2 |


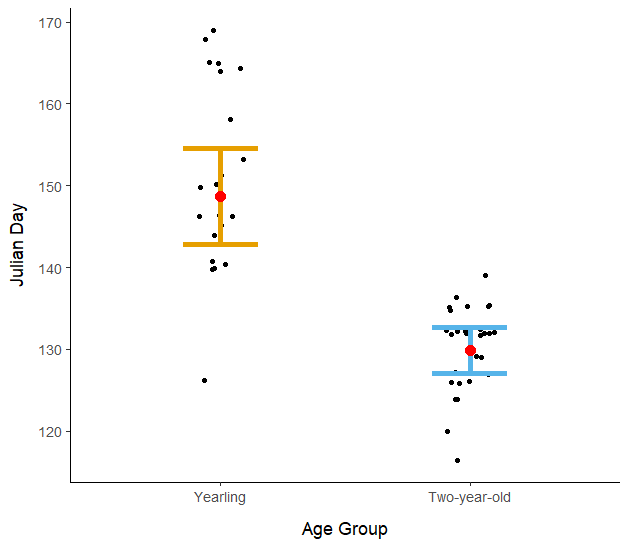


Figure S1: Predicted breakup date range in relation to the age of the offspring (1.5-year-old yearlings = orange and 2.5-year-old two-year-olds = blue) in southcentral Sweden (2007-2021). The prediction plot is based on the most parsimonious model (Table S1), only including the age of the offspring as a fixed variable and a nested random effect of mother- and family-ID. The black dots represent the raw data (*n* = 49).

**Appendix 2 - moveVis animations including male presence**

Male Family Breakup 1

Video 1: Family (mother and 1 yearling) with an adult male present at the time of breakup.

Male Family Breakup 2

Video 2: Family (mother and 3 two-year-olds) with an adult male present at the time of breakup.
